# Correction: Borges et al. The Roles of Potassium and Calcium Currents in the Bistable Firing Transition. *Brain Sci.* 2023, *13*, 1347

**DOI:** 10.3390/brainsci14080808

**Published:** 2024-08-12

**Authors:** Fernando S. Borges, Paulo R. Protachevicz, Diogo L. M. Souza, Conrado F. Bittencourt, Enrique C. Gabrick, Lucas E. Bentivoglio, José D. Szezech, Antonio M. Batista, Iberê L. Caldas, Salvador Dura-Bernal, Rodrigo F. O. Pena

**Affiliations:** 1 Department of Physiology and Pharmacology, State University of New York Downstate Health Sciences University, Brooklyn, NY 11203, USA; 2 Center for Mathematics, Computation and Cognition, Federal University of ABC, São Bernardo do Campo 09606-045, Brazil; 3 Institute of Physics, University of São Paulo, São Paulo 05508-090, Brazil; 4 Graduate Program in Science, State University of Ponta Grossa, Ponta Grossa 84010-330, Brazil; 5 Department of Mathematics and Statistics, State University of Ponta Grossa, Ponta Grossa 84030-900, Brazil; 6 Center for Biomedical Imaging and Neuromodulation, The Nathan S. Kline Institute for Psychiatric Research, Orangeburg, NY 10962, USA; 7 Department of Biological Sciences, Florida Atlantic University, Jupiter, FL 33458, USA; 8 Stiles-Nicholson Brain Institute, Florida Atlantic University, Jupiter, FL 33458, USA; 9 Federated Department of Biological Sciences, New Jersey Institute of Technology and Rutgers University, Newark, NJ 07102, USA

**Funding Updated**

In the original publication [1], a funder, the National Science Foundation, DMS-1608077 and IOS-2002863, was not included. The updated funding part appears below.

**Funding:** The authors acknowledge the financial support of the São Paulo Research Foundation (FAPESP, Brazil) (Grants N. 2018/03211-6, 2020/04624-2, 2021/12232-0, 2022/13761-9), Fundação Araucária, CNPq, Coordenação de Aperfeiçoamento de Pessoal de Nível Superior—Brasil (CAPES), and NIH U24EB028998. The authors also acknowledge NSF 2002971-DIOS and DMS-1608077.

**Additional Affiliation**

In the published publication, there was a missing affiliation for Rodrigo F. O. Pena. The newly added affiliation appears below.

9 Federated Department of Biological Sciences, New Jersey Institute of Technology and Rutgers University, Newark, NJ 07102, USA

The authors state that the scientific conclusions are unaffected. This correction was approved by the Academic Editor. The original publication has also been updated.

## References

[1] Borges F.S., Protachevicz P.R., Souza D.L.M., Bittencourt C.F., Gabrick E.C., Bentivoglio L.E., Szezech J.D., Batista A.M., Caldas I.L., Dura-Bernal S. (2023). The Roles of Potassium and Calcium Currents in the Bistable Firing Transition. Brain Sci..

