# Supplementary material for: The Roles of Potassium and Calcium Currents in the Bistable Firing Transition
Source: Brain Sci. 2023 Sep 20;13(9):1347. doi: 10.3390/brainsci13091347 (PMC10527161; doi:10.3390/brainsci13091347)
Supplement: Supplementary file 1 [file brainsci-13-01347-s001.zip › brainsci-2585300-supplementary.pdf]

1. Neuron dynamics

Figure S1 illustrates the biophysical properties of neurons and their dependence on the ionic conductance of the slow potassium M-current with the additional presence of calcium currents. The frequency of action potentials increases with the input amplitude, as shown by the three exemplar voltage traces (Fig. S1A). For input amplitudes of $I = 40$ pA, the neuron presents a single spike after ≈ 150 ms from the start of stimulus (Fig. S1B). The second spike occurs for $I = 47$ pA, where the first frequency F is obtained by $1/ISI_1$. Regular spiking behavior is observed for $I > 160$ pA (Fig. S1C). In Fig. S1D we show the minimum value of I where $F > 0$ for $g_T = g_L = 0$ (gray circles), the black line is a curve fit

$$I = 82.394 + 1559.2 * g_M + 1837.3 * (g_M)^2. \quad (1)$$

For $g_T = 0.4$ mS/cm^2 and $g_T = 0.2$ mS/cm^2 (orange squares) the fitted curve is

$$I = 74.655 + 1672.5 * g_M + 1719.6 * (g_M)^2. \quad (2)$$

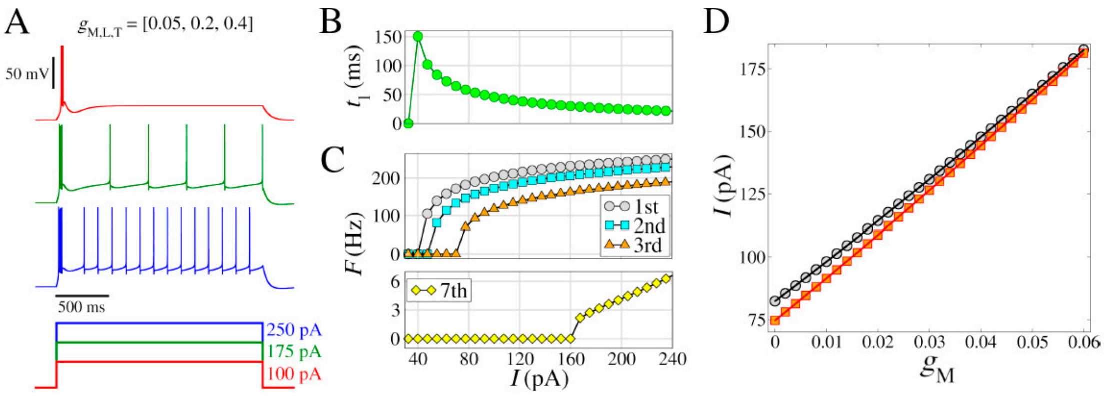

Figure S1. Model of regular spiking neuron, with I_M, I_T, and I_L. (A) (Top) Voltage traces with different amplitudes of the depolarizing pulses (bottom). **(B)** Time to first spike (t_1) as a function of the injected current (amplitude of the pulse I). **(C)** Frequency-current curves (F/I), where the instantaneous firing rate (inverse of the inter-spike interval) is represented as a function of I. The curves indicated by different colors correspond to the 1st, the 2nd, the 3rd, and the 7th spike in the train. **(D)** Transition where $F > 0$ for $g_T = 0$ and $g_L = 0$ (black line) and $g_T = 0.4$ mS/cm^2 and $g_L = 0.2$ mS/cm^2 (red line). Other parameters are $L = d =$ 96.0 μm, $g_{leak} = 0.01$ mS/cm^2, $E_{leak} = -85.0$ mV, $g_{Na} = 50$ mS/cm2, $V_T = -55.0$ mV, $g_{Kd} = 5$ mS/cm^2, $\tau_{max} = 1000$ ms, and $g_M = 0.05$ mS/cm^2.

Figure S2 presents colored (g_M, g_L)-diagrams for F and CV when $I = 200$ pA (A–B) and $I = 300$ pA (C–D). The region in which sustained bursts occur (CV > 1.0) is lower for $I = 300$ pA than for $I = 200$ pA. The firing rate (F) increases when $I = 300$ pA for all (g_M, g_L)-diagram.

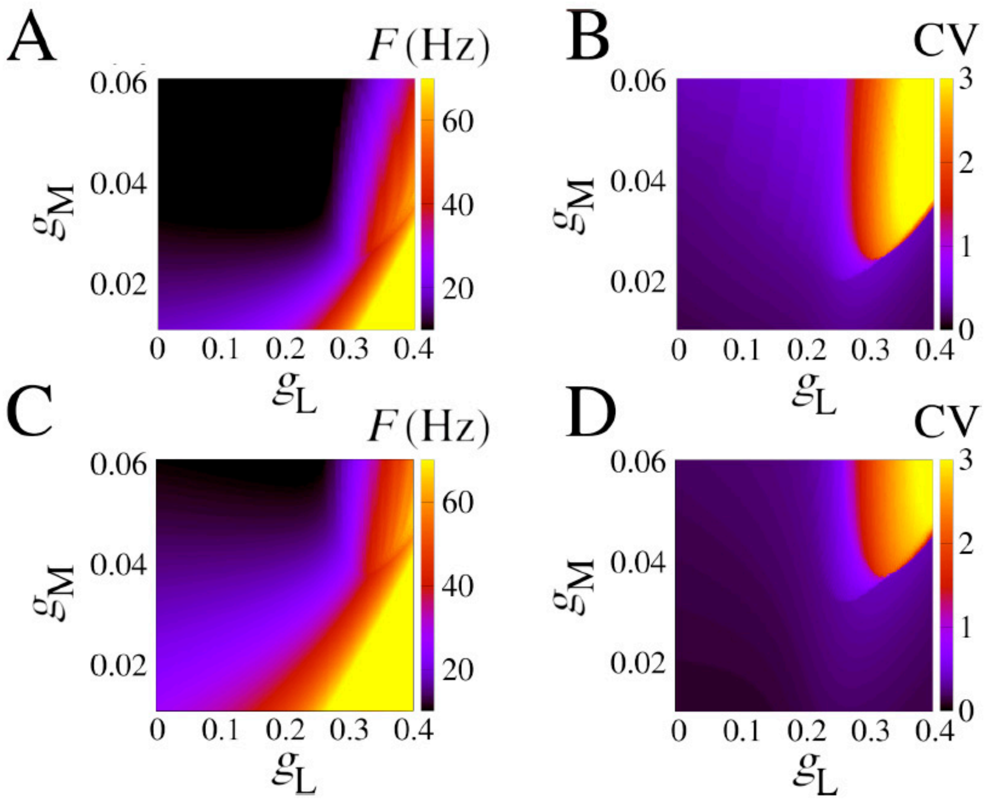

Figure S2. Firing pattern for diferent I_M and I_L conductances. **(A)** Firing rate in colored (g_M, g_L)-diagram for I = 200 pA. **(B)** The same as **A** for the CV. **(C)** Firing rate in colored (g_M, g_L)-diagram for I = 300 pA. **(D)** The same as **C** for the CV. Other parameters are the same as Fig. S1 with g_T = 0.4 mS/cm^2.

2. Neuron network

In Figure S3 the values of CV, F, and R are shown at the top, middle, and bottom, respectively, in dependence on the constant current and the chemical synaptic conductance (I,g_{syn}) with respect to different values of $g_{M,L,T}$. The bistable parameter region is identified in white and separates the burst-synchronized region from the asynchronous ones. The high-threshold calcium conductance (g_L) allows for transition at lower values of g_{syn} (Fig. S3C). Moreover, the burst-synchronized area is bigger in this case. In contrast, low-threshold calcium promotes the opposite effect by slightly increasing the value of g_{syn} necessary to observe a transition (compare Fig. S3A to B and C to D).

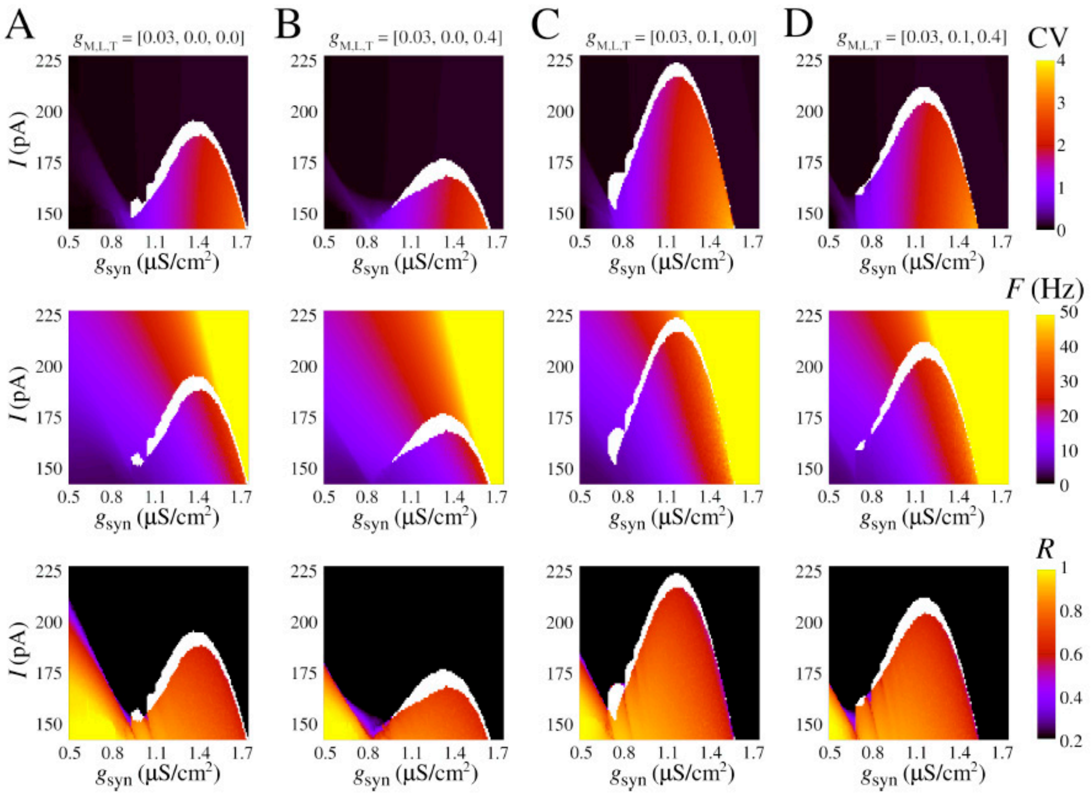

Figure S3. Changes in network firing pattern with input current (I) and chemical synaptic conductance (g_{syn}). (A-D) CV (top), *F* (middle), and *R* (bottom) for different combinations of $g_{M,L,T}$ (see values atop).

The dynamics of the membrane potential (V), the total synaptic current (I_{syn}), and the total ionic current ($I_{ionic} = I_{Na} + I_K + I_M + I_L + I_T$) are shown in Fig. S4 for an example neuron in the network. The parameters used are the same as in Fig. S3D with g_{syn} = 0.95 μS/cm^2 and I = 190 pA, therefore, in the bistable region. For these parameters, when synchronous initial conditions were considered, burst synchronization is observed (Fig. S4A–E). On the other hand, assynchronous spikes occur with asynchronous initial conditions (Fig. S4F–J). Furthermore, for better understanding of each ionic channel type in neuronal activity, the ionic currents were divided into positive (%I_{out}) and negative (%I_{in}) contributions and normalized. Figure S4D shows the outward ionic current fractions (I_{leak}, I_K, I_M) in burst synchronization. The inward ionic current fractions (I_{Na}, I_L, I_T) are shown in Fig. S4E. Figures S4 I–J are shown the same as Fig. S4D–E for the asynchronous pattern. I_K, I_L, and I_T have a stronger contribution during action potentials, where the fraction of I_L is approximately 50 times higher than I_T.

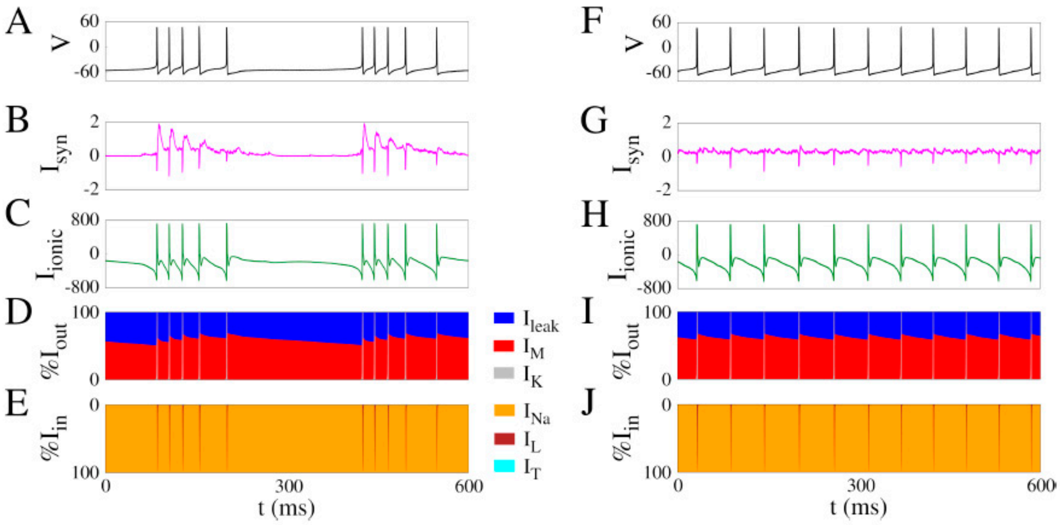

Figure S4. Dynamics of ionic currents in a neuron during the bistable regime. **(A)** Voltage traces (V, in mV). **(B)** Total synaptic current (I_{syn}, in μA/cm²). **(C)** Total ionic current (I_{ionic}, in μA/cm²). **(D)** Fraction of outward ionic currents (%I_{out}). **(E)** Fraction of inward ionic currents (%I_{in}). The parameters are the same of Fig. S3D, in bistable region, with g_{syn} = 0.95 μS/cm² and I = 190 pA. For the (A–E) simulations synchronous initial conditions were considered. **(F–J)** The same than (A–E) but with asynchronous initial conditions.
